# Impact of Sprinting and Dribbling on Shoulder Joint and Pushrim Kinetics in Wheelchair Basketball Athletes

**DOI:** 10.3389/fresc.2022.863093

**Published:** 2022-06-02

**Authors:** Félix Chénier, Ilona Alberca, Dany H. Gagnon, Arnaud Faupin

**Affiliations:** ^1^Mobility and Adaptive Sports Research Lab, Department of Physical Activity Science, Université du Québec à Montréal (UQAM), Montreal, QC, Canada; ^2^Centre for Interdisciplinary Research in Rehabilitation of Greater Montreal (CRIR), Montreal, QC, Canada; ^3^Université de Toulon, Impact de l'Activité Physique sur la Santé (UR IAPS n°201723207F), Campus de La Garde, Toulon, France; ^4^School of Rehabilitation, Université de Montréal, Faculty of Medicine, Montreal, QC, Canada

**Keywords:** wheelchair sports, adaptive sports, performance, shoulder dynamics, biomechanics

## Abstract

**Background:**

While wheelchair basketball is one of the most popular Paralympic sports, it eventually causes shoulder problems and pain in many athletes. However, shoulder kinetics has never been assessed during propulsion in wheelchair basketball. This study analyzes the impact of sprinting and dribbling on pushrim and shoulder kinetics in terms of external forces and net muscular moments.

**Methods:**

A group of 10 experienced wheelchair basketball athletes with various classifications performed four, 9-m sprints on a basketball court using classic synchronous propulsion, and four sprints while dribbling forward. Pushrim and shoulder kinetics were calculated by inverse dynamics, using a motion capture device and instrumented wheels.

**Findings:**

Sprinting was associated to peak shoulder load from 13 to 346% higher than in previous studies on standard wheelchair propulsion in most force/moment components. Compared to sprinting without a ball, dribbling reduced the speed, the peak external forces in the anterior and medial direction at the shoulder, and the peak net shoulder moment of internal rotation.

**Interpretation:**

The high shoulder load calculated during both sprinting and dribbling should be considered during training sessions to avoid overloading the shoulder. Dribbling generally reduced the shoulder load, which suggests that propelling while dribbling does not put the shoulder at more risk of musculoskeletal disorders than sprinting.

## Introduction

Adaptive sports offer many important benefits for people with disorders and disabilities, such as decreasing the risk of cardiovascular disease, improving general health and enhancing quality of life (1). Among the various adaptive sports available, wheelchair basketball (WB) is one of the most popular and is the most advanced in terms of organization, standardization and training quality (2–4). Each player is assigned a classification according to their functional ability. In Canada, where classification closely follows the International Wheelchair Basketball Federation (IWBF) but also allows able-bodied athletes to play, this classification ranges from 1 point (players with the least ability) to 4.5 points (minimal to no impairment).

This sport, which is very similar to its abled-bodied counterpart, contains intermittent phases of high intensity combining wheelchair maneuvers and ball handling. However, it is possible that such high intensity may be detrimental to the athletes' musculoskeletal integrity. In everyday mobility, propelling a standard manual wheelchair (MW) is considered in itself a high intensity activity and causes musculoskeletal disorders (MSD) in half of all MW users, especially at the shoulder (5–7).

It is still unclear whether playing WB puts the musculoskeletal system at higher risk compared to standard wheelchair propulsion. Finley and Rogers (6) found no difference in the occurrence of shoulder pain between athletic and non-athletic MW users. Wheelchair sports could have a protective effect by delaying the onset of symptoms (8), but these observations contradict those of Mateus (9) where 17 of 25 participants who reported pain during the last year attributed their pain to WB. Furthermore, Akbar et al. (10) reported that 76% of athletes who perform overhead sports such as WB have rotator cuff impairments, compared to 25% in non-athletes.

Nevertheless, elite wheelchair athletes are subject to high shoulder injury rates (11). In WB, the most reported disorders are rotator cuff impingement or a tear, biceps tendinopathy, and acromioclavicular joint pathology (12, 13). While it is unclear if these injuries are due to overhead movements, to wheelchair maneuvering, or (probably) to a combination of both, Mercer et al. (14) found in a previous study on the propulsion of standard MW, that specific components of shoulder load are associated to shoulder disorders:

1) increased external glenohumeral forces in posterior and lateral directions, and increased internal moments in flexion and adduction, are related to a higher prevalence of coracoacromial ligament edema and/or thickening that may lead to subacromial impingement and rotator cuff tear;
2) increased external glenohumeral forces in superior direction and increased internal moment in external rotation, are related to increased signs of symptomatic shoulder pathology.

To date, measurement of shoulder joint kinetics in WB athletes has been performed only in non-ecological conditions such as isokinetic testing (15). Therefore, the aim of this exploratory work is to measure the shoulder kinetics in WB athletes during the propulsion of a sports wheelchair in ecological conditions, and to compare these measurements to previous measurements in standard MW propulsion. This work focuses on two mobility aspects of WB: sprinting and dribbling. We assessed the impact of these tasks on both pushrim and shoulder kinetics, and more precisely: on the three components of the pushrim forces (tangential, radial, and mediolateral) and the propulsive moment, to obtain insight on the efficiency of the applied force during the complete push phase, on shoulder dynamics, to evaluate the effect of sprinting and dribbling in relation to the association between shoulder load and MSD described by Mercer et al. (14).

We hypothesized that shoulder load would be higher in sports wheelchair sprinting than in previous studies on standard MW propulsion. Moreover, in light of our previous results (16) where dribbling reduced the mean propulsive moments compared to sprinting, we hypothesized that dribbling would generally decrease the shoulder load.

## Methods

### Participants

Ten WB athletes participated in this experiment. To be included, athletes could not have a current or recent ( ≤ 3 months) injury or pain that could interfere with their ability to carry out the tasks. The experimental protocol was approved by the Institutional Research Ethics Committee of Université du Québec à Montréal (UQAM) (certificate #CIEREH 2879_e_2018). This work is based on the same data as presented in Chénier et al. (16), except that participant #9 in the first study was replaced by participant #4 due to a problem with the motion capture device. Participant demographics are provided in Table 1, where participants are ordered by classification and by years of experience in WB.

**Table 1:** Participant demographics.

**Participant**	**Sex**	**Age**	**Dominant**	**Disorder**	**Height**	**Weight**	**BMI**	**Experience**	**Classification**	**Belt/**	**Wheel**	**Wheel**
	**(M/F)**	**(years)**	**limb (R, L)**		**(m)**	**Weight (kg)**	**(kg/m^2^)**	**(years)**	**(1.0–4.5)**	**strap**	**size (in)**	**camber (deg)**
1	F	31	R	SCI T6-A	1.60	61	23.8	3	1.0	torso thigh	25	20
2	M	60	R	SCI D6-D7-A	1.83	71	21.2	6	1.0	torso thigh	26	16
3	M	29	L	CP	1.68	60	21.3	10	1.0	thigh	25	19
4	M	40	R	SCI T12-A	1.75	66	21.6	12	1.0	torso thigh	25	22
5	M	34	R	SCI T7-A	1.50	73	32.4	10	1.5	thigh	26	18
6	M	33	R	SCI T10-A	1.76	95	30.7	1.5	2.0	thjgh	26	19
7	M	32	R	MD	1.73	52	17.4	6	2.0	thigh legs	25	22
8	M	23	R	SD	1.63	58	21.8	11	2.0	thigh	26	17
9	F	30	R	ND	1.61	62	23.9	3	4.5	thigh	26	20
10	M	24	R	ND	1.78	78	24.6	16	4.5	thigh	26	20
Mean (SD)	7×M 3×F	33.6 (10.5)	9×R 1×L	/	1.69 (0.10)	67.6 (12.3)	23.9 (4.5)	7.9 (4.7)	2.1 (1.4)	/	4×25in 6×26in	19.3 (1.9)

*SCI, Spinal Cord Injury; CP, Cerebral Palsy; MD, Muscular Dystrophy; SD, Spastic Dysplasia; ND, Non-disabled*.

### Tasks

After a personal 5-min warm-up, every participant performed 9-m sprints at maximal speed in a straight line from a stopped position on a wooden basketball court. Participants were asked to propel synchronously, with both arms pushing at the same time, in two conditions:

1) Classic Propulsion (CP), during four sprints, without a ball.
2) Dribble Propulsion (DP), during four sprints, where they were instructed to forward dribble. After two acceleration pushes, they had to push the ball forward, give one push on the wheels, recover the ball on the rebound, then place the ball on their knees, as described in Chénier et al. (16). They were asked to repeat this sequence until they had completed the 9-m distance.

A total of eight sprints was recorded: 2 conditions × 2 sides (right/left) × 2 repetitions. The order of the sprints was randomized, and participants were allowed to rest for a self-selected duration between trials.

### Instrumentation

#### Kinetics

Participants used their own sports wheelchair equipped bilaterally with two instrumented wheels (SmartWheel). A wheel size of 25 or 26 inches was selected based on the participant's wheelchair. The instrumented wheels measured the propulsion forces and moments in 3D around the wheel hubs at 240 Hz. These wheels have a weight and moment of inertia of approximately 4.9 kg and 0.15 kg·m^2^ (17). To limit the added resistance due to their increased weight, the SmartWheels' standard solid tires were switched to inflatable tires and fully inflated to 110 PSI.

#### Kinematics

An optoelectronic system consisting of 14 cameras (Prime13, Optitrack) was used to measure the participants' kinematics unilaterally. The cameras were arranged to build an acquisition volume that covered the entire sprint. The following landmarks were recorded at 120 Hz: second metacarpal distal heads, center of the hand, ulnar and radial styloid processes, lateral and medial elbow epicondyles, acromion, C7, T12, and both rear wheel centers. Landmarks that could not be followed directly due to occlusion (e.g., rear wheel center of the opposite side, medial elbow epicondyle) were reconstructed using rigid clusters of three to four markers affixed on the wheelchair, arms, and forearms. The position of the second metacarpal distal heads was not measured directly but was calculated using the styloid processes and hand markers. The rear wheel camber was measured using static kinematic acquisitions where different points of the wheels were probed and expressed relative to the wheelchair.

### Data Processing

#### Kinetics

The dynamic offsets in the measured pushrim forces and moments due to the wheel camber were canceled as described in Chénier et al. (18). Synchronization between kinetics and kinematics was done at the beginning of each recording, by gently impacting the instrumented pushrim with a stick instrumented with a reflective marker. This impact was identified as a simultaneous event in both instruments: as a force spike in the kinetic data, and as a sudden stop of the marker's motion in the kinematic data.

#### Kinematics

**Figure 1:**
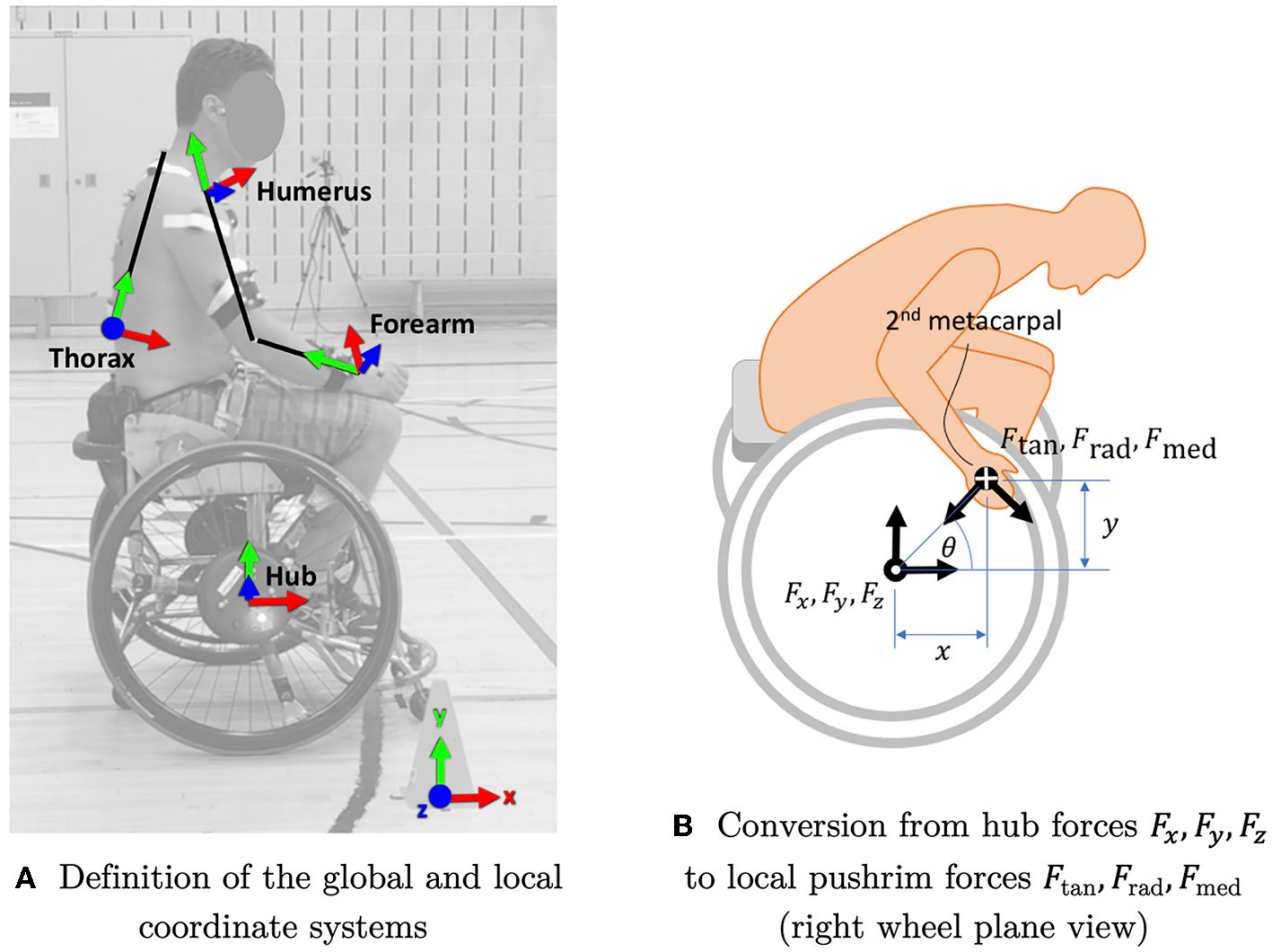
**(A,B)** Coordinate system definitions.

Marker positions were filtered at 10 Hz using a second-order, no-lag Butterworth filter. The definition of the coordinate system is provided in Figure 1A. The forearm and humerus coordinate systems were defined following the recommendations of the International Society of Biomechanics (ISB) (19), using both elbow epicondyles and both styloid processes, and approximating the glenohumeral joint by the acromion. Because of the flexed position adopted by some participants, the thorax could only be defined by markers in the back (T12 and C7). Since propulsion was synchronous, we considered that the thorax was not axially rotated, and therefore the y axis of the thorax was defined as the line from T12 to C7, and the yz plane of the thorax was defined by its y axis and the wheelchair's mediolateral axis. The coordinate systems of the wheel hubs were defined at the hub centers with their y and z axes inclined according to the wheel camber. All left side recordings were mirrored across the wheelchair mediolateral axis, and all subsequent data processing was considered right sided.

#### Inverse Dynamics

A generic inverse dynamics method composed of four segments (wheel, forearm+hand, arm, thorax) was used to iteratively calculate the shoulder joint kinetics from the wheel's hub to the second metacarpal distal head, then to the elbow center and finally to the shoulder joint (20). Inertial characteristics (mass, moments of inertia) were personalized based on each participant's mass, sex and segments length, using inertial data compiled by Winter [(21), chap. 4. Anthropometry].

#### Push Selection

For all conditions, pushes 1 and 2 were considered to be transitional and were discarded from the analysis. For the CP condition, all pushes after push 3 (included) were analyzed. For the DP condition, pushes performed while the ball was in the air, and deemed valid as described in section 3.2 were analyzed.

#### Outcome Variables

Speed was defined as the speed reached at the end of the fourth push and was calculated based on the wheel angles, using a 131-point, first-order derivative Savitzky-Golay filter (22).

The total pushrim force and the three pushrim force components were calculated by converting the pushrim forces *F*_*x*_, *F*_*y*_ and *F*_*z*_ and moment *M*_*z*_ (measured in the non-rotating hub coordinate system), to the point of force application estimated by the position of the second metacarpal distal head as shown in Figure 1B:

- Total pushrim force: $F_{tot}=\sqrt{F_{x}^{2}+F_{y}^{2}+F_{z}^{2}}$
- Tangential pushrim force: *F*_tan_ = *F*_*x*_sin *θ* − *F*_*y*_cos *θ*
- Radial pushrim force: *F*_*rad*_ = −*F*_*x*_cos *θ* − *F*_*y*_sin *θ*
- Medial pushrim force: *F*_*med*_ = − *F*_*z*_
- Propulsive moment: *M*_*prop*_ = *M*_*z*_

where *θ* is an angle in the wheel plane, between a horizontal line and a line from the wheel center to the projected second metacarpal distal head.

Shoulder forces and moments were expressed in the thorax coordinate system. The reported forces are external, i.e., a superior shoulder force means that the external reaction force pushes the humeral head upward relative to the thorax. The reported moments are internal and relate to the net muscular action at the shoulder joint.

For each analyzed push, the following outcome variables were calculated:

- Pushrim kinetics: peak values of *F*_*tot*_, *F*_tan_, −*F*_tan_, *F*_*rad*_, −*F*_*rad*_, *F*_*med*_, −*F*_*med*_, *M*_*prop*_ and −*M*_*prop*_;
- External shoulder forces: peak values of anterior, posterior, superior, inferior, lateral and medial forces.
- Internal shoulder moments: peak values of flexion, extension, adduction, abduction, internal rotation, and external rotation moments.

#### Statistical Analysis

For each outcome variable, data normality of the difference between both conditions was verified using a Shapiro-Wilk test with α = 0.05. For data where normality was confirmed, parametric tests (paired *t*-tests) with α = 0.05 were used to test for the mean difference between both propulsion conditions. Due to the exploratory nature of this work, significance thresholds were not corrected for multiple comparisons. The effect size was reported for every comparison using:


$$\begin{array}{l} d=\frac{\text{mean}\left({\text{x}}_{\text{DP}}\right)-mean\left(x_{\text{CP}}\right)}{\text{s}.\text{d}.\left({\text{x}}_{\text{CP}}\right)} \end{array}$$

and was interpreted using Cohen's recommendation: small (*d* = 0.2), moderate (*d* = 0.5) and large (*d* = 0.8) (23). For data that fail the Shapiro-Wilk normality test, non-parametric tests (Wilcoxon's signed rank tests) were used instead, and the effect size was calculated using the rank-biserial correlation.

For each condition, we also plotted typical profiles for the pushrim forces, shoulder forces and shoulder moments during the push. To reduce both the intra-participant and inter-participant variability, each assessed variable *x* was first time-normalized from −25 to 125% of the push, and then amplitude-normalized using:


$$\begin{array}{l} normalized\left(x\left(\%push\right)\right)=x\left(\%push\right)\times \frac{\left(\bar{{\text{A}}_{\text{p}-\text{p}}}\right)}{{\text{A}}_{\text{p}-\text{p}}} \end{array}$$

where *A*_p−p_ is the peak-to-peak amplitude of *x* for a given push cycle, and $\bar{A_{\text{p}-\text{p}}}$ is the averaged *A*_p−p_ over every push of a given condition (CP, DP).

All calculations were performed with Python/SciPy using Kinetics Toolkit (24). Statistics were calculated using JASP 0.14.1.

#### Comparison to Other Studies

The calculated shoulder kinetics were compared to results from 10 studies from 2001 and up that used a similar method (inverse dynamics with rigid bodies) to calculate the shoulder load during standard MW propulsion. To avoid comparing too different conditions, we only included results from non-elderly wheelchair users, without upper-limb impairment, who propelled a real wheelchair (as opposed to an integrated, custom ergometer). This resulted in a total of 10 studies, in which the participants propelled on rollers, treadmills or an ascending ramp at speeds from 0.8 to 2.2 m/s (14, 25–32).

## Results

### Outcome Variables

Table 2 shows the outcome variables and their comparison between both conditions. Individual results are also available as graphs in Supplementary Material.

**Table 2:** Comparison of outcome measures between both conditions.

	**CP**		**DP**		**Diff**		***p***	**d**	**np**	**Standard MW^a^**.
Speed (m/s)	2.57	(0.32)	2.39	(0.31)	−0.18	(0.16)	**0.007**	**−1.11**		
**Peak pushrim kinetics**
Total force *F*_*tot*_ (*N*)	215.8	(46.7)	202.0	(44.3)	−13.7	(24.2)	0.11	**−0.57**		
Forward tangential force *F*_tan_ (*N*)	146.9	(41.9)	140.1	(32.5)	−6.7	(19.1)	0.30	−0.35		
Negative tangential force −*F*_tan_ (*N*)	21.0	(7.4)	15.5	(6.4)	−5.6	(4.9)	**0.01**	**−0.86**		
Inward radial force *F*_*rad*_ (*N*)	160.3	(37.1)	133.7	(49.3)	−26.6	(34.5)	**0.04**	**−0.77**		
Outward radial force −*F*_*rad*_ (*N*)	15.8	(11.6)	21.5	(11.6)	5.6	(5.2)	**0.01**	**1.09**		
Medial force *F*_*med*_ (*N*)	86.4	(35.7)	90.1	(32.7)	3.7	(10.8)	0.31	0.34		
Lateral force −*F*_*med*_ (*N*)	11.2	(7.7)	6.3	(4.6)	−4.9	(4.2)	**0.005**	**−1.17**		
Propulsion moment *M*_*prop*_ (Nm)	37.2	(9.5)	35.80	(8.35)	−1.42	(4.40)	0.70	−0.16	^*^	
Braking moment −*M*_*prop*_ (Nm)	5.1	(1.7)	4.25	(1.52)	−0.82	(1.38)	0.08	**−0.64**	^*^	
**Peak shoulder forces**
Anterior (*N*)	118.4	(24.9)	91.0	(27.9)	−27.4	(21.8)	**0.003**	**−1.25**		5–50
Posterior (*N*)	171.8	(42.4)	157.6	(35.3)	−14.2	(27.4)	0.16	**−0.53**		27–92
Superior (*N*)	60.1	(19.1)	64.3	(15.4)	4.2	(14.2)	0.38	0.30		−16–108
Inferior (*N*)	79.8	(22.8)	88.1	(44.4)	8.3	(32.7)	0.45	0.25		−33–58
Lateral (*N*)	66.6	(30.3)	61.5	(28.3)	−5.1	(11.0)	0.18	**−0.46**		7–50
Medial (*N*)	66.5	(23.4)	48.0	(20.7)	−18.6	(11.7)	**<0.001**	**−1.59**		7–15
**Peak shoulder moments**
Flexion (Nm)	65.3	(17.8)	58.78	(13.4)	−6.49	(9.8)	0.07	**−0.66**		6–40
Extension (Nm)	31.0	(8.5)	24.61	(11.4)	−6.40	(15.2)	0.22	−0.42		5–17
Adduction (Nm)	40.6	(13.0)	40.72	(15.6)	0.11	(6.2)	0.56	0.24		0–31
Abduction (Nm)	30.3	(12.8)	21.03	(9.8)	−9.24	(10.8)	0.85	0.00		0–12
Internal rotation (Nm)	23.8	(11.7)	19.02	(12.9)	−4.74	(6.5)	**0.05**	**−0.73**		0–21
External rotation (Nm)	41.3	(17.3)	38.83	(15.0)	−2.48	(6.8)	0.28	−0.36		0–21

*Parentheses, standard deviation; d, effect size; np, non-parametric test.*

*Bold and underlined p-values indicate **p < 0.05** and **p < 0.01**.*

*Bold and underlined d-values indicate **moderate** (0.5) to **large** (>0.8) effect sizes.*

* *Non-parametric test.*

a *Peak shoulder kinetics ranges from previous studies on standard MW propulsion on treadmill or rollers from 0.8 to 2.2 m/s (14, 25–33)*.

#### Pushrim Kinetics

In both CP and DP, the tangential and inward radial forces were the two most important force components. A braking moment and a negative tangential force were observed. Dribbling had no effect on the propulsive components of the pushrim kinetics (i.e., the peak tangential force and peak propulsive moment). However, dribbling generally reduced the peak negative tangential force in 9 of the 10 participants (−5.6 *N*, −27%, *p* = 0.01, *d* < −0.8). Dribbling also mainly reduced the non-propulsive force components: the peak lateral force decreased in 9 participants (−4.9 *N*, −44%, *p* < 0.01, *d* < −0.8), and the peak inward force decreased in 8 participants (−26.6 *N*, −17%, *p* = 0.04, *d* = −0.77), although at the expense of an increase in peak outward force in 7 participants (+5.6 *N*, +35%, *p* = 0.01, *d* > 0.8).

#### Shoulder Forces

In the following sections, each main force/moment component is reported and compared to its maximal counterpart from the 10 studies indicated in section 3.4.7. The main external shoulder force was in the posterior direction (172 N), which is 87% higher than the same component measured by Kloosterman et al. (28) with 11 wheelchair users who propelled at 0.9 m/s on a treadmill (92 N). The second highest shoulder force component was in the anterior directions (118 N), which is 136% higher than the same component measured by Gil-Agudo et al. (26) with 16 wheelchair users who propelled at 1.1 m/s on a treadmill (50 N). Compared to sprinting, dribbling reduced the peak anterior force in 9 participants (−27.4 *N*, −23%, *p* < 0.01, *d* < −0.8), and the peak medial force 9 participants (−18.6 *N*, −30%, *p* < 0.01, *d* < −0.8).

#### Shoulder Moments

The main net joint moment was in flexion (65 Nm), which is 64% higher than the same component measured by Sabick et al. (33) with 16 wheelchair users who propelled on a 20:1 ascending ramp (40 Nm). The second main moments were both in adduction and external rotation. Adduction (41 Nm) was 31% higher than the same component measured by Koontz et al. (29) with 27 individual with SCI who propelled at 1.8 m/s on rollers (21 Nm). External rotation (41 Nm) was 101% higher than the same component measured by Collinger et al. (25) in a multisite study with 61 wheelchair users who propelled at 1.8 m/s on rollers (21 Nm). Compared to sprinting, the main effect of dribbling on shoulder moments was in the transverse and sagittal planes. Dribbling reduced the peak internal rotation moment in 7 participants (−4.74 *Nm*, −20%, *p* = 0.05, *d* = −073).

### Kinetic Profiles

**Figure 2:**
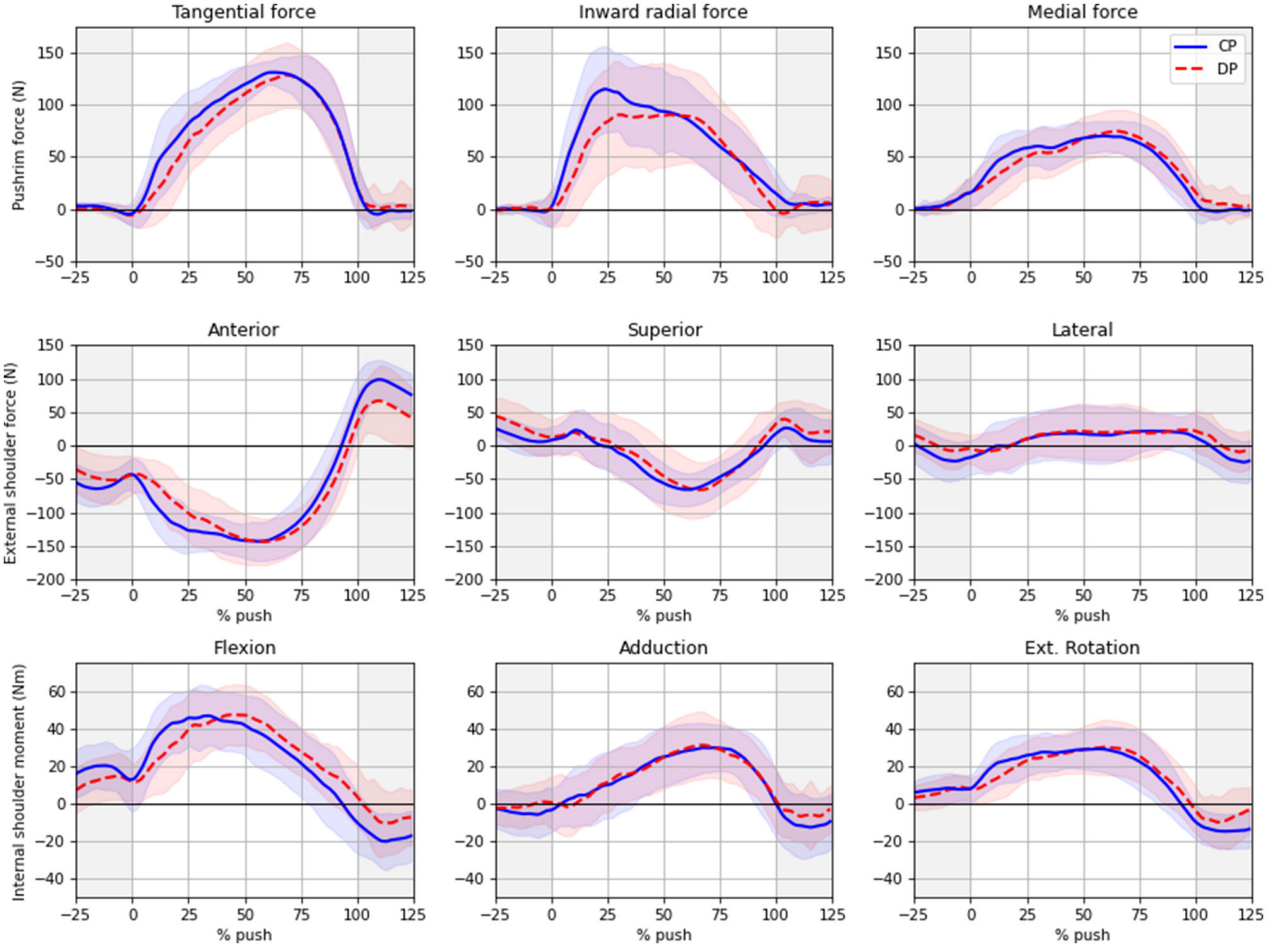
Kinetic profiles during CP and DP conditions.

Figure 2 shows the typical profile for the pushrim forces, shoulder forces and shoulder moments from −25 to 125% of the push. Both conditions have similar profiles. At the shoulder, external forces in posterior, inferior and lateral direction, and net moments in flexion, adduction and external rotation were observed during the push. The inverse was observed after releasing the pushrims. In DP, we observed a decreased inward radial pushrim force, which peaks at about 25% of the push in the CP condition compared to a plateau between 30 and 65% of the push in the DP condition. We also observed a decreased anterior shoulder force and a decreased shoulder moment of internal rotation during the transition from push to recovery, at about 110% of the push. Finally, we observed a decreased slope for each pushrim force component, a delayed anterior shoulder force and a delayed shoulder moment of flexion.

## Discussion

The aim of this work was to assess the effect of sprinting and dribbling using a sports wheelchair on the different components of the pushrim and shoulder kinetics. Compared to previous studies on standard MW propulsion, the shoulder load is much higher, independently of the CP or DP condition. Obviously, difference in speeds between these studies and ours most probably account for these large differences. However, while the participants in our study propelled only 0.4 m/s faster than in Mulroy et al. (31), with 2.6 m/s compared to 2.2 m/s, the posterior shoulder force was 130% higher (172 vs. 75 Nm) and the flexion moment was 170% higher (65 vs. 24 Nm). Apart from the superior and inferior shoulder force that varies a lot between studies, every shoulder force of moment component was 13% to 346% higher than its highest counterpart in every other study. In addition to the wheelchair's geometry and user's position that are different between standard and sport wheelchairs, these large differences in shoulder kinetics may be explained by two reasons. The first reason is that every of these other studies were performed during continuous propulsion on rollers or treadmill, whereas our study was conducted on the ground. While our conditions were more ecologically valid, the athletes did not completely reach their maximal velocity after only two pushes; the remaining acceleration requires higher propulsion moments. The second reason relates to the limbs' inertia. Since the speed was higher in our study, the joint forces required for accelerating and decelerating the limbs were also higher. The effect of these inertial components can be seen in Figure 2, where immediately after the push, no force is applied on the wheel, but important shoulder forces and moments can still be observed, especially in the anterior shoulder force and in the shoulder moments of flexion, adduction and external rotation.

The values found in this work are generally high and may be worrisome. For example, the peak posterior shoulder force was 172 N, compared to 42 N in Mercer et al. (14) who correlated such high values to an increased risk of coracoacromial ligament disorder. Moreover, the peak shoulder moment of external rotation was 41 N, compared to 9 N in Mercer et al. (14) who correlated such high values to symptomatic shoulder pathology. This raises a flag on the intensity of propelling in WB compared to everyday propulsion. However, in WB, half of the game time is spent coasting or resting, and a rather small percentage of the time is performed sprinting (9%) or dribbling (<1%) (34). Thus, we believe that the causes of shoulder disorders could not only be associated with sprinting or dribbling, but most probably to a combination of tasks such as accelerating, challenging/handling the ball, and sprinting. However, this high load should be considered when planning training sessions to avoid overloading the shoulder.

When comparing CP and DP, dribbling reduced every peak force value except the positive tangential and medial forces. Dribbling also reduced the peak negative tangential forces. This combined reduction in peak force components is viewed as a beneficial change in terms of push efficiency. However, these differences may be attributable to the reduced speed observed during dribbling: similar relationships between speed and pushrim kinetics have been observed in a study by Kwarciak et al. (35) where 54 participants with paraplegia who propelled their own wheelchair on rollers increased the amplitude and the number of occurrences of negative moments as speed increased. In terms of shoulder kinetics, we expected that dribbling would decrease the shoulder load. We indeed observed a reduction in the peak posterior shoulder force, such a component being associated with coracoacromial ligament edema or thickening in standard MW propulsion (14). Since dribbling was not associated with other specific kinetic components related to shoulder disorders, this suggests that propelling while dribbling may be less detrimental to the shoulder joint than sprinting.

In this work, we chose to refer to the shoulder moments in the thorax reference frame to be consistent with Mercer et al. (14). However, special care must be taken in interpreting the results in this reference frame, especially shoulder moments of internal/external rotation. When the arms are not elevated, the reported moments of rotation in either the thorax or humeral reference frame are similar because the humeral and thorax longitudinal axes are nearly coincident. However, when the arm is more elevated like it is in sports wheelchair propulsion, the reported shoulder rotation moments may include significant crosstalk (moments from other axes). For example, for a 90-degree abduction, a moment reported in the thorax reference frame as an external rotation would be better understood as a moment of horizontal abduction. This example highlights the difficulty of comparing shoulder load between tasks that are kinematically different. Currently, there is no consensus on the best way to report shoulder kinetics. Some authors (including those of this work) reported shoulder kinetics in the thorax reference frame (14). Others reported the forces in the thorax frame but the moments in the humeral frame (25), while others used four components instead of three, with three standard anatomical axes associated with the thorax reference frame, and an additional axis (the humerus longitudinal axis) to express humeral rotation moments (29, 36). Research is still needed to define what axes are the best axes to report shoulder kinetics as a function of the studied task.

Among the limits of the study, we note the limited number of participants and their variety of disorders and classifications. However, since the observed differences between both conditions and between previous literature were generally large, we believe that this work allowed much needed insight to be gained on the impact of wheelchair propulsion in WB on shoulder load. Another limitation is the evaluation of the associated risks of shoulder disorders using the work of Mercer et al. (14) who assessed these risks for standard MW propulsion on rollers, not for sports wheelchair propulsion on a basketball court. It is therefore important to consider these comparisons as indicative and not as a direct relationship between propulsion and specific shoulder disorders. Finally, using SmartWheel instrumented wheels increased the rolling resistance and wheelchair inertia due to their added weight. We however limited this effect by using fully inflatable tires instead of the standard solid Smart Wheel tires.

As highlighted in this work, the differences between sprinting and dribbling on shoulder load seem much lower than the differences between everyday propulsion and sports propulsion. Consequently, we believe that including dribbling sessions in addition to sprinting sessions during training should not be riskier for the shoulder, which supports our previous conclusions based on spatiotemporal and generic pushrim kinetic parameters (16). Future work should reproduce a similar analysis to other tasks found in WB, such as accelerating, changing direction, and challenging and handling the ball, which would increase our understanding of the risks of MSD associated with WB.

## Data Availability Statement

The raw data supporting the conclusions of this article will be made available by the authors, without undue reservation.

## Ethics Statement

The studies involving human participants were reviewed and approved by Université du Québec à Montréal's Comité institutionnel d'éthique de la recherche avec des êtres humains (CIEREH). The patients/participants provided their written informed consent to participate in this study.

## Author Contributions

FC contributed to the study design, to the data processing and is the main author of the manuscript. IA contributed to data processing. DG and AF contributed to the study design. All authors contributed to manuscript revision, read, and approved the submitted version.

## Funding

This work was funded by the Fonds de recherche du Québec via the Société Inclusive intersectoral initiative.

## Conflict of Interest

The authors declare that the research was conducted in the absence of any commercial or financial relationships that could be construed as a potential conflict of interest.

## Publisher's Note

All claims expressed in this article are solely those of the authors and do not necessarily represent those of their affiliated organizations, or those of the publisher, the editors and the reviewers. Any product that may be evaluated in this article, or claim that may be made by its manufacturer, is not guaranteed or endorsed by the publisher.
