# Supplementary material for: Impact of Sprinting and Dribbling on Shoulder Joint and Pushrim Kinetics in Wheelchair Basketball Athletes
Source: Front Rehabil Sci. 2022 Jun 2;3:863093. doi: 10.3389/fresc.2022.863093 (PMC9397776; doi:10.3389/fresc.2022.863093)
Supplement: Supplementary file 1 [file Image_1.PDF]

Speed, peak pushrim kinetics and peak shoulder kinetics per participant

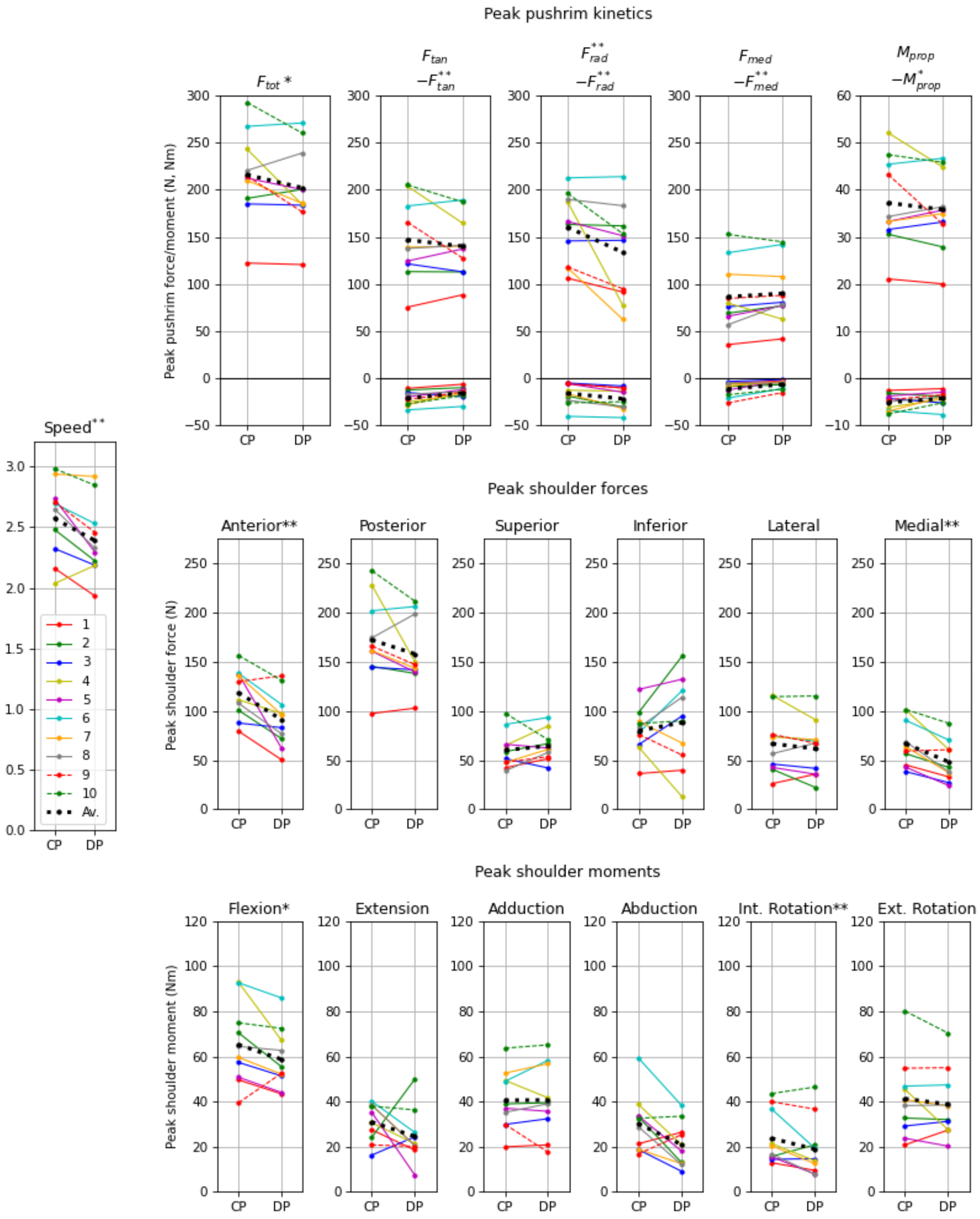

Moderate (*) and large (**) effect sizes are marked with asterisks.
